# Isothermal Recombinase Polymerase amplification (RPA) of *Schistosoma haematobium* DNA and oligochromatographic lateral flow detection

**DOI:** 10.1186/s13071-015-1055-3

**Published:** 2015-09-04

**Authors:** A. Rosser, D. Rollinson, M. Forrest, B L Webster

**Affiliations:** Faculty of Infectious and Tropical Diseases, London School of Hygiene and Tropical Medicine, London, UK; Department of Life Sciences, Natural History Museum, London, UK; TwistDX Ltd, Cambridge, UK

**Keywords:** *S. haematobium*, Diagnostics, Urine, DNA amplification, Isothermal, RPA

## Abstract

**Background:**

Accurate diagnosis of urogenital schistosomiasis is vital for surveillance/control programs. Amplification of schistosome DNA in urine by PCR is sensitive and specific but requires infrastructure, financial resources and skilled personnel, often not available in endemic areas. Recombinase Polymerase Amplification (RPA) is an isothermal DNA amplification/detection technology that is simple, rapid, portable and needs few resources.

**Findings:**

Here a *Schistosoma haematobium* RPA assay was developed and adapted so that DNA amplicons could be detected using oligochromatographic Lateral Flow (LF) strips. The assay successfully amplified *S. haematobium* DNA at 30–45 °C in 10 mins and was sensitive to a lower limit of 100 fg of DNA. The assay was also successful with the addition of crude urine, up to 5 % of the total reaction volume. Cross amplification occurred with other schistosome species but not with other common urine microorganisms.

**Conclusion:**

The LF-RPA assay developed here can amplify and detect low levels of *S. haematobium* DNA. Reactions are rapid, require low temperatures and positive reactions are interpreted using lateral flow strips, reducing the need for infrastructure and resources. This together with an ability to withstand inhibitors within urine makes RPA a promising technology for further development as a molecular diagnostic tool for urogenital schistosomiasis.

## Findings

### Introduction

*Schistosoma haematobium* is one of three major human schistosome species and causes >110 million cases of urogenital schistosomiasis throughout Africa, parts of the Middle East, Madagascar, the Indian Ocean Islands and more recently Corsica. Infection can result in, hematuria, bladder wall pathology, hydronephrosis leading to severe kidney disease [1, 2] and bladder cancer [3]; in addition, an estimated 16 million women suffer from female genital schistosomiasis (FGS), which can cause complications in relation to fertility and HIV transmission [4].

Sensitive and specific diagnostic tests are critical for the development and success of schistosomiasis control and elimination programs. They not only enable the accurate diagnosis and treatment of individual patients but also provide accurate surveillance data necessary to assess the effectiveness of control interventions and to detect resurgence [5]. Moreover, as a control program achieves success, low infection intensity is common increasing the need for improved diagnostic sensitivity and specificity to prevent false negative diagnosis [6–10].

Various modalities for the diagnosis and surveillance of urogenital schistosomiasis are available but none exhibit optimal performance characteristics for sensitive and specific Point-Of-Care (POC) diagnosis [7–12]. Molecular diagnostics that target and amplify schistosome DNA from within urine and stool samples potentially offer high sensitivity and specificity [7, 8, 13, 14]. However, these methods are costly, do require significant laboratory infrastructure and technical ability, which can restrict their use in endemic field settings [15].

Recombinase polymerase amplification (RPA) is an isothermal DNA amplification technology offering several advantages over other DNA amplification methods especially in terms of its application in more basic laboratory settings in the field. The recombinase polymerase reaction is specific, robust and can take place at an ambient temperature [16, 17]. DNA amplification can be detected by oligochromatographic lateral flow (LF) strips or real time fluorescence, which offers detection flexibility and utility in endemic settings [18]. Here we describe a pilot project investigating the viability of RPA to amplify the *S. haematobium* Dra1 DNA region [19]. The assays specificity and sensitivity were investigated together with its ability to withstand PCR inhibitors found within crude urine.

### RPA Assay development

Suitable (30–35 bp, GC content 30–70 %) RPA primers were designed (using Primer3 (http://primer3.ut.ee/)), to target 110–120 bp of the *S. haematobium* Dra1 (www.ncbi.nlm.nih.gov/genbank, GenBank: DQ157698.1) DNA region.

**Fig. 1:**
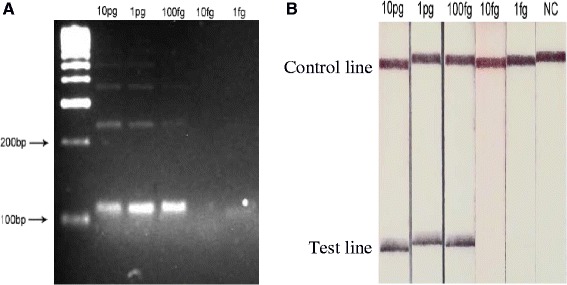
**a** Agarose gel image of the Dra1 RPA amplicons. Low limit of detection can be seen at 100 fg of *S. haematobium* gDNA. **b** LF strips showing the detection of the Dra LF-RPA amplicons. Lower limit of detection can be seen at 100 fg of *S. haematobium* gDNA. NC = Negative control. The LF-RPA oligonucleotides consisted of a forward primer, a specialised 6-FAM labelled oligonucleotide probe and a reverse biotin labelled primer. Upon successful binding to the complementary gDNA target, amplification ensues resulting in the formation of a double-labelled amplicon. When run on an oligochromatographic LF strip, the amplicon binds to anti-FAM antibodies and antibody labelled gold colloid nanoparticles in the running buffer bind to the biotin antigen resulting in a semi quantitative colour change. The LF strip also has a control line to test for reaction failure

Combinations of the different RPA forward and reverse primers were tested for their ability to amplify the Dra1 repeat region using the TwistAmp Basic kit (TwistDx, UK), with slight modifications to the manufacturer’s protocol. 1X rehydration buffer and dH_2_O was added to the dry enzyme pellet and thoroughly mixed. 10 μM of each forward and reverse primer was added together with 1 ng of template *S. haematobium* gDNA (Senegal strain from the Schistosomiasis Collection at the Natural History Museum (SCAN) [19]) and the reaction was initiated by addition of 280 mM of magnesium acetate. Reactions were incubated at 37 °C with constant shaking at 300 rpm in a Minitron shaker (Infors, Switzerland) for 20 min. The reactions were purified with a QIAquick PCR Purification Kit (Qiagen, USA) and then visualised on a 4 % agarose gel. The primer pair, Dra-RPA-F1 + R1 (Table 1), demonstrated the best target amplification (111 bp), producing amplicon bands of increasing size representing multiples of the original target sequence [20] (Fig. 1). The sensitivity of the assay was evaluated with a detection limit of 100 fg of DNA observed (Fig. 1a).

**Table 1:** Primers / probe sequence design for the Dra1 RPA and LF assays

Assay	Name	Sequence (5′-3′)
RPA	Dra1-F1	ATCTCACCTATCAGACGAAACAAAGAAAAT
	Dra1-R1	AATATGAAACAATTTTCACAACGATACGAC
LF-RPA	Dra1-F1-LF	ATCTCACCTATCAGACGAAACAAAGAAAAT
	Dra1-R1-LF	(Z)AATATGAAACAATTTTCACAACGATACGAC
	Dra1 LF probe	(Y)AATTGTTGGTGGAAGTGCCTGTTTCGCAAT(H)TCTCCGGAATGGTTG(3)

**Features**: **Z** = Biotin label; **Y** = 6-carboxyfluorescein (FAM) label; **H** = abasic tetrahydrofuran (THF) residue; **3** = C3 spacer. All primers were designed according to the instructions from TwistDx (http://www.twistdx.co.uk). The Dra1 LF probe was designed with a recommended length of 46–52 bp with at least 30 bp placed 5′ to the abasic tetrahydrofuran (THF) residue (**H**). The RPA and LF RPA primers are the same but with a 5′ modification on the reverse primer (Dra1-R1-LF) for the LF RPA assay

### Dra1 LF-RPA assay development

The Dra1_RPA_F1 + R1 primers were adapted for the LF-RPA assay by labelling of the reverse primer with a 5′ biotin residue and an internal RPA LF probe (Dra1 LF probe) was designed according to the TwistDX guidelines (Table 1). 5 μM of each LF primer (Dra1-LF-F1 + R1), 5 μM of the Dra1 LF probe (10 μM), 0.25 M betaine (Sigma Aldrich, UK) was mixed with 1 x rehydration buffer, dH2O and was added to the reaction pellet of the TwistAmp nfo kit (TwistDX, UK). 1 ng of the template *S. haematobium* DNA was added and the reaction was initiated by addition of 280 mM of magnesium acetate. Reactions were incubated at 37 °C in a Minitron shaker at 300 rpm for 20 min. After incubation, 1 μl of the amplification was diluted in the running buffer (Milenia Biotec, Germany) and 10 μl placed on the sample pad of a Genline Hybridetect −1 LF strip (Milenia Biotec, Germany). The strips were placed vertically into separate tubes containing 100 μl of running buffer and incubated at room temperature with the final result read at 5 min.

**Fig. 2:**
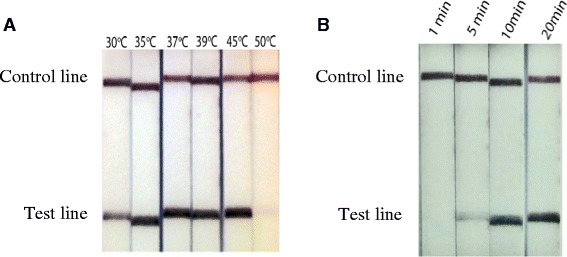
LF strips showing Dra1 LF-RPA amplicon detection at different reaction temperatures (**a**) and times (**b**)

Detection of the Dra1 LF-RPA amplicons was observed as a positive test line on the LF strips (Fig. 1b). Faint test bands in the negative controls were sometimes observed if the LF strips were left for too long before being read, creating false positives. Increasing the amplicon dilution in the running buffer to a ratio of 1 in 100 from the recommended 1 in 50 abrogated this problem. The sensitivity of Dra1 LF-RPA assay was evaluated with a limit of detection observed at 100 fg of DNA (Fig. 1b). The effect of varying reaction temperatures and times were also assessed. The assay was found to operate over a temperature range of 30–45 °C, with reduced amplification at lower temperatures and no amplification at 50 °C (Fig. 2a). Amplification was detectable after as little as 5 min of incubation and optimal amplification was reached at 10 min (Fig. 2b).

### Effect of crude urine

Increasing volumes (0.31 μl, 0.63 μl, 1.25 μl and 2.50 μl) (Table 2) of donor urine were spiked with 100 fg and also 10 pg of *S. haematobium* gDNA before being added to the Dra1 LF-RPA reactions. Each reaction volume was adjusted to a final volume of 25 μl with H_2_0 so that the percentage of crude urine within the reactions ranged from 1 to 10 %. With 100 fg of *S. haematobium* gDNA the addition of 1.25 % urine had no impact upon amplification efficacy. A marked reduction in amplification efficacy was qualitatively noted at 2.5 % and 5 % of total reaction volume and no amplification was visible at 10 %. In contrast gDNA levels of 10 pg increased the assays efficacy with significant amplification being detected even at 10 % of total reaction volume of urine.

**Table 2:** Results showing the impact of crude urine on the efficacy of the Dra1 RPA-LF assay and subsequent LF detection. All reactions were performed at 37 °C for 20 mins

Measure of crude urine in each Dra1 RPA-LF reaction		Dra1 RPA-LF efficacy	
Volume of crude urine (μl)	% of crude urine	Amount of *S. haematobium* gDNA	
		10 pg	100 fg
0.31	1.25 %	+++	+++
0.63	2.5 %	+++	++
1.25	5 %	+++	+
2.50	10 %	+++	-

The number of + refers to the qualitative signal on the LF test line. +++ = very strong and - = no trace

### Specificity

Cross amplification was observed with 1 pg of *S. curassoni* and *S. bovis* DNA and at a higher limit of 100 pg for *S. mansoni* (samples supplied by SCAN [19]). No cross amplification was seen with *Escherichia coli*, *Klebsiella pneumoniae*, *Proteus mirabilis* (cultures supplied by the Microbiology Department, University Hospitals of Leicester) and human gDNA (www.promega.com).

### Discussion

The detection of species specific gDNA from urine is the sensitive and specific current marker of true *S. haematobium* infection [21]. RPA is an alternative gDNA based diagnostic tool that overcomes several of the obstacles encountered by traditional gDNA based diagnostics [16–18], particularly suiting it to Point-Of-Care (POC) use [22]. All reagents are readily available lyophilized with the main RPA reagents provided in a single dried pellet, which simplifies assay preparation and allows easy transportation and long-term storage at room temperature. DNA amplification is quick, requiring low ambient temperatures and this together with the LF detection system is very simple and portable to use, with results easily interpreted by personnel with little training [16–18].

Here, RPA was successfully developed to amplify the Dra1 DNA region of *S. haematobium* and amplification success was easily detected by a semi quantitative colourimetric change on LF strips. Amplification was detectable after only 5 min of incubation and at 30 °C, thus incubation using human body heat, ambient environmental temperatures in endemic areas or battery powered devices would suffice, removing the need for external power supplies and infrastructure [22].

The current LF-RPA sensitivity was lower than existing Dra1 amplification assays [21]. However, theoretically RPA can detect single template DNA copies thus there is scope for further LF-RPA optimisation and development to increase the sensitivity of the assay to achieve that achieved with RT-PCR and LAMP [21]. RPA offers degrees of tolerance to inhibitors, such as those found in urine [17] suggesting direct testing of untreated urine from infected people will be feasible. The RPA’s reaction mechanisms and chemistry means that total purification of total DNA from samples is not required [17], reducing the need for expensive, technical and time-consuming sample DNA preparation / purification, enhancing its feasibility in a POC situation [23]. The current cost per test is high at approximately $5 (reaction + detection) however, RPA is a newly developed technique and prices are likely to decrease in the future while availability and throughput will increase. For example centrifugal microfluidic cartridges that can split a single reaction to enable testing of eight individual samples have been developed [22] reducing the cost to $0.6.

The Dra1 LF-RPA specificity to schistosome DNA was high with no cross-reaction with Enterobacteria or human gDNA. As found previously with RT-PCR [24], RPA Dra1 cross-amplification with *S. mansoni* was 1000 fold less sensitive than for *S. haematobium* but as predicted cross-amplification was successful with *S. curassoni* and *S. bovis,* both members of the *S. haematobium* group, known to possess the Dra1 repeat sequence [25]. Thus there is potential to utilise this assay and / or develop alternative species-specific assays for the identification of other *S. haematobium*-group species infections in livestock and humans.

In conclusion, the Dra1 LF-RPA assay developed here provides a robust *S. haematobium* DNA amplification system offering advantages over currently available DNA diagnostic systems; requiring minimal infra structure, equipment, technical support, sample preparation and is tolerant to inhibitors in crude urine. With further development this assay has potential for POC diagnosis of urogenital schistosomiasis in endemic field settings.

## Abbreviations

RPA: Recombinase polymerase amplification
LF: Lateral flow
gDNA: Genomic deoxyribonucleic acid
POC: Point-of-care
PCR: Polymerase chain reaction
RT-PCR: Real time PCR
